# Endemic *Amorphophallus* (Araceae) from Madagascar: a revised key, a new species and molecular phylogeny

**DOI:** 10.1186/1999-3110-55-2

**Published:** 2014-01-14

**Authors:** Wilbert L A Hetterscheid, Cyrille Claudel

**Affiliations:** 1 Von Gimborn Arboretum, Velperengh 13, Doorn, 3941 BZ The Netherlands; 2 Biocentre Klein Flottbek & Botanical Garden, Ohnhorststrasse 18, Hamburg, 22609 Germany

**Keywords:** *Amorphophallus hildebrandtii*, *Amorphophallus perrieri*, *Amorphophallus*, Araceae, Endemic, Madagascar, New species, Phylogeny

## Abstract

**Background:**

Since the revision of *Amorphophallus* of Madagascar (Bot Jahrb Syst 121(1):1–17, 1999) several additional new species have been described. The recent discovery of another new species promted the preparation of a revised key as well as the description of the new species. *Amorphophallus hildebrandtii*, never restudied since its analysis by Engler in 1881, has been refound and restudied. Meanwhile molecular phylogenetic studies have provided new insights in the relationships of the endemic Madagascan species.

**Results:**

The new species *Amorphophallus perrieri* is described. A new revised key to the endemic Amorphophallus species of Madagascar is provided. An emended description of *A. hildebrandtii* is provided. A molecular phylogeny of the endemic Madagascan species of *Amorphophallus* is provided.

**Conlusions:**

The enigmatic character of a very short spadix in *A. hildebrandtii* has been confirmed, after it was thought for many years that it was artificially shorter in the holotype specimen than in nature. This was suggested by the fact that the spadix of the holotype is broken. The monophyletic character of the Madagascan endemic species clade remained unchallenged after analysis including all new species discovered recently, incl. the new species presented in this paper.

**Electronic supplementary material:**

The online version of this article (doi:10.1186/1999-3110-55-2) contains supplementary material, which is available to authorized users.

## Background

The earliest publication of an *Amorphophallus* on Madagascar was by Engler (1881) when he published, under the now generic synonym, *Hydrosme hildebrandtii* Engl., based on a specimen collected by J.M. Hildebrandt (#3161). After transfer to *Amorphophallus* by Engler (1911) *A. hildebrandtii* (Engl.) Engl. was long believed to be the only species endemic on Madagascar. Bogner (1972, 1975) mentions specimens found on the mainland of Madagascar and identified them as *A. hildebrandtii* (Engl.) Engl. & Gehrm. One such plant was figured by Hetterscheid & Ittenbach (1996, Figures 114 and 115) also using the name *A. hildebrandtii*. In preparation of a revision of the African *Amorphophallus* species by Ittenbach, the first author mentioned his doubts as to the identifications of mainland *Amorphophallus* plants as *A. hildebrandtii*. The field books of Hildebrandt were subsequently researched by Josef Bogner and it was found that the type locality of *A. hildebrandtii* had to be on the island of Nosy Be (not mentioned by Engler, nor indicated on the type specimen). In a subsequent publication (Hetterscheid et al. 1999) the mainland specimens of *Amorphophallus* earlier thought to represent *A. hildebrandtii* were described as two new species (*A. ankarana* Hett., Ittenb. & Bogner and *A. taurostigma* Ittenbach, Hett. & Bogner). In the same paper a morphologically more deviating new species was also introduced, *A. antsingyensis* Bogner, Hett. & Ittenbach.

However, it was still at the time considered that the short appendix of the type of *A. hildebrandtii* was an artifact, but this turned out not to be the case when photographs by Stephan Vogel revealed an *Amorphophallus* seen at the Lokobé Reserve on Nosy Be, just off the NW coast of Madagascar. This plant showed exactly the short appendix for *A. hildebrandtii* described by Engler. The photographs were published in Ittenbach’s revision of the African species of *Amorphophallus* (Ittenbach 2003). More recently, Olaf Pronk also succeeded in finding *A. hildebrandtii* on Nosy Be. In cultivation some of them flowered and showed the unique morphology of spathe and spadix of this species. Pictures of this are shown below, and an updated description of the species is provided.

In 2003, Josef Bogner published a small-statured new species as *A. mangelsdorffii* Bogner. This species differs from all others on Madagascar in flowering alongside the leaf late in the rainy season.

Hetterscheid and Mangelsdorff (2006) published two new species from Madagascar, *A. erythrorrhachis* Hett., O. Pronk & R. Kaufmann and *A. andranogidroensis* Hett. & Mangelsdorff.

**Figure 1:**
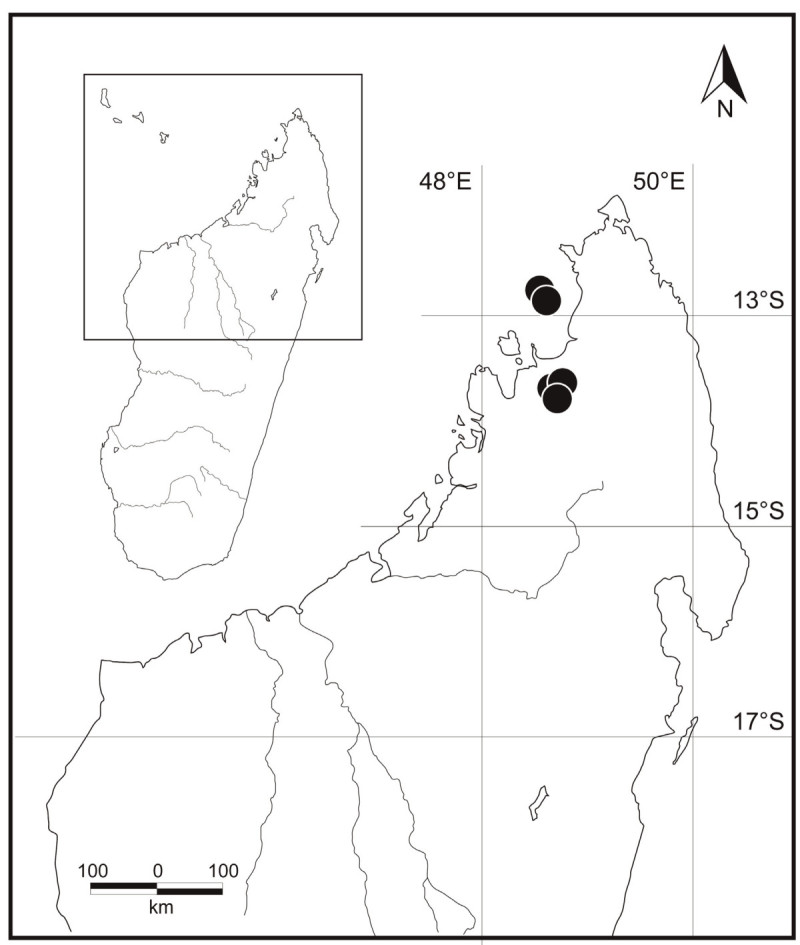
**Distribution of** ***Amorphophallus perrieri*** **(black dots) in the Antsiranana Province, Madagascar.**

Recently a new species was discovered independently by several people, in Kalabenono (N. Madagascar), and on the island of Nosy Mitsio just off the coast of N. Madagascar by Olaf Pronk, and on Nosy Mitsio and Nosy Ankarea by Greg Wahlert (Figure 1). It subsequently turned out that a specimen of this same species had been collected in 1932 on Nosy Mitsio by J.M.H.A. Perrier de la Bâthie, and deposited in the Paris herbarium. Ittenbach noticed this specimen too and annotated it as “*A. hildebrandtii* subsp. *bathiesii*, nov. subsp.” but did not publish this name. The new species is now described here as *A. perrieri* Hett. & Wahlert in honour of Perrier de la Bâthie.

This brings the total of known endemic *Amorphophallus* species on Madagascar to eight. The ninth species occurring on Madagascar is the introduced *A. paeoniifolius* (Dennst.) Nicolson. A new key is provided.

## Methods

Plants of *A. perrieri* were grown by G. Wahlert at the University of Utah (USA) under tropical greenhouse conditions until flowering, as well as by the first author in the greenhouse of the former botanical gardens of Wageningen University (Netherlands). Inflorescences were studied in detail and photographed and preserved dried or in spirit collection.

For molecular analysis DNA of the markers ITS1, Flint2, rbcL and matK was sequenced and aligned (data not shown) resulting in a data matrix containing 3970 characters of which 3848 characters were constant, 95 variable characters were parsimony-uninformative and 27 were parsimony-informative. A maximum-likelihood tree was generated using MEGA version 5 (Tamura et al. 2011). Missing data and gaps were deleted; further parameters used are given in the Appendix.

## Results and discussion

### Key to the amorphophallus species of Madagascar

**Figure 2:**
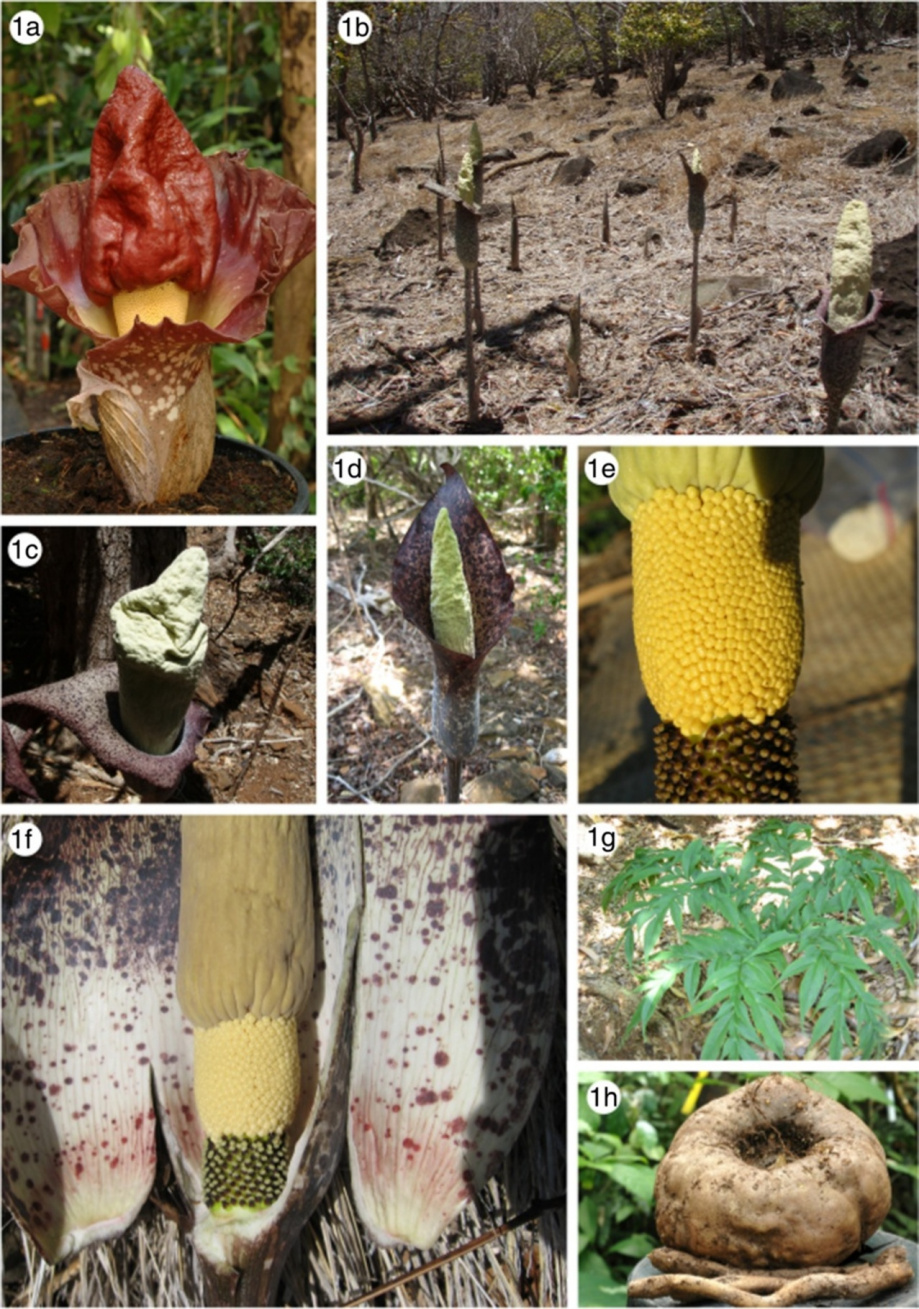
**Morphology of Amorphophallus species of Madagascar.** Amorphophallus paeoniifolius, inflorescence **(a)**; Amorphophallus perrieri, population on Nosy Mitsio **(b)**; Amorphophallus perrieri, inflorescence **(c)**; Amorphophallus perrieri, inflorescence **(d)**; Amorphophallus perrieri, detail of base of the spadix **(e)**; Amorphophallus perrieri, inflorescence cut open, showing the inner spathe and base of spadix **(f)**; Amorphophallus perrieri, leaf blade from above **(g)**; Amorphophallus perrieri, tuber, the two rhizomatous offsets broken off **(h)**. **b-h** by G. Wahlert (USA).

1a Peduncle much shorter than spathe, appendix rounded or conical, petiole rough, tuber with distinctly raised, annulate rootscars...........................*A. paeoniifolius* (Figure 2a)

1b Peduncle longer than spathe, appendix narrowly or broadly elongate-conical, petiole smooth, tuber surface smooth.........................................................................................2

2a Appendix with upper part corrugated, often strongly so............*A. perrieri* **sp. nov.** (Figure 2a-h)

2b Appendix with upper part smooth..................................................................3

3a Spadix distinctly longer than spathe...................................................4

**Figure 3:**
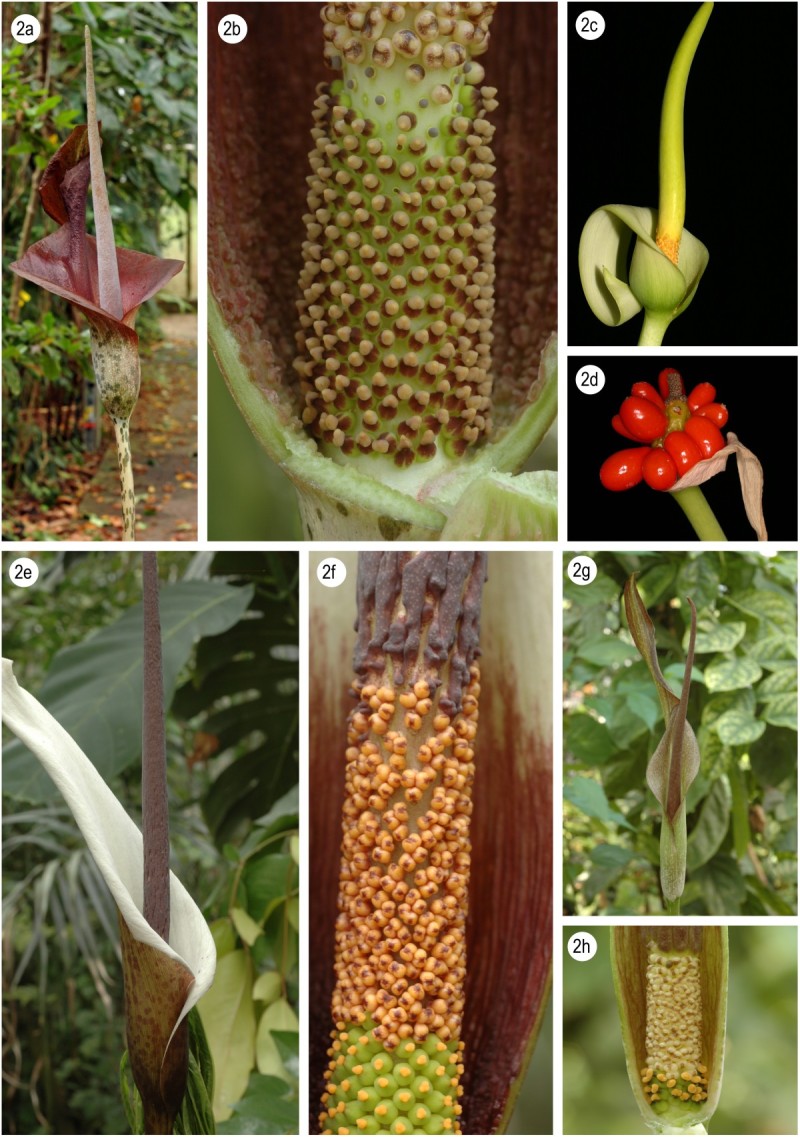
**Morphology of Amorphophallus species of Madagascar.** Amorphophallus taurostigma, inflorescence **(a)**; Amorphophallus taurostigma, detail of the spadix base **(b)**; Amorphophallus mangelsdorffii, inflorescence **(c)**; Amorphophallus mangelsdorffii, fruiting **(d)**; Amorphophallus ankarana, inflorescence **(e)**;Amorphophallus ankarana, detail the spadix base **(f)**; Amorphophallus andranogidroensis, inflorescence **(g)**; Amorphophallus andranogidroensis, detail of the spadix base **(h)**. **c, d** by R.M. Mangelsdorff (Germany).

4a Style present, long, thin…......................*A. taurostigma* (Figure 3a, b)

4b Style (near-)absent……………….....................……………..5

5a Leaf and inflorescence found at the same time (late rainy season)................................…*A. mangelsdorffii* (Figure 3c, d)

5b Inflorescence always preceding the leaf (beginning of rainy season)…..............................................................…..6

Stigma shallowly 2-3-lobed, at most 0.5 - 1.3 mm in diam., tuber disciform, offsets sessile, globose..............................................…*A. ankarana* (Figure 3e, f)

Stigma entire or very shallowly bilobed, 1.3 - 1.5 mm in diam., tuber depressed globose, offsets very long rhizomatous...........*A. andranogidroensis* (Figure 3g, h)

3b Spadix distinctly shorter than or equal to the spathe..............................7

**Figure 4:**
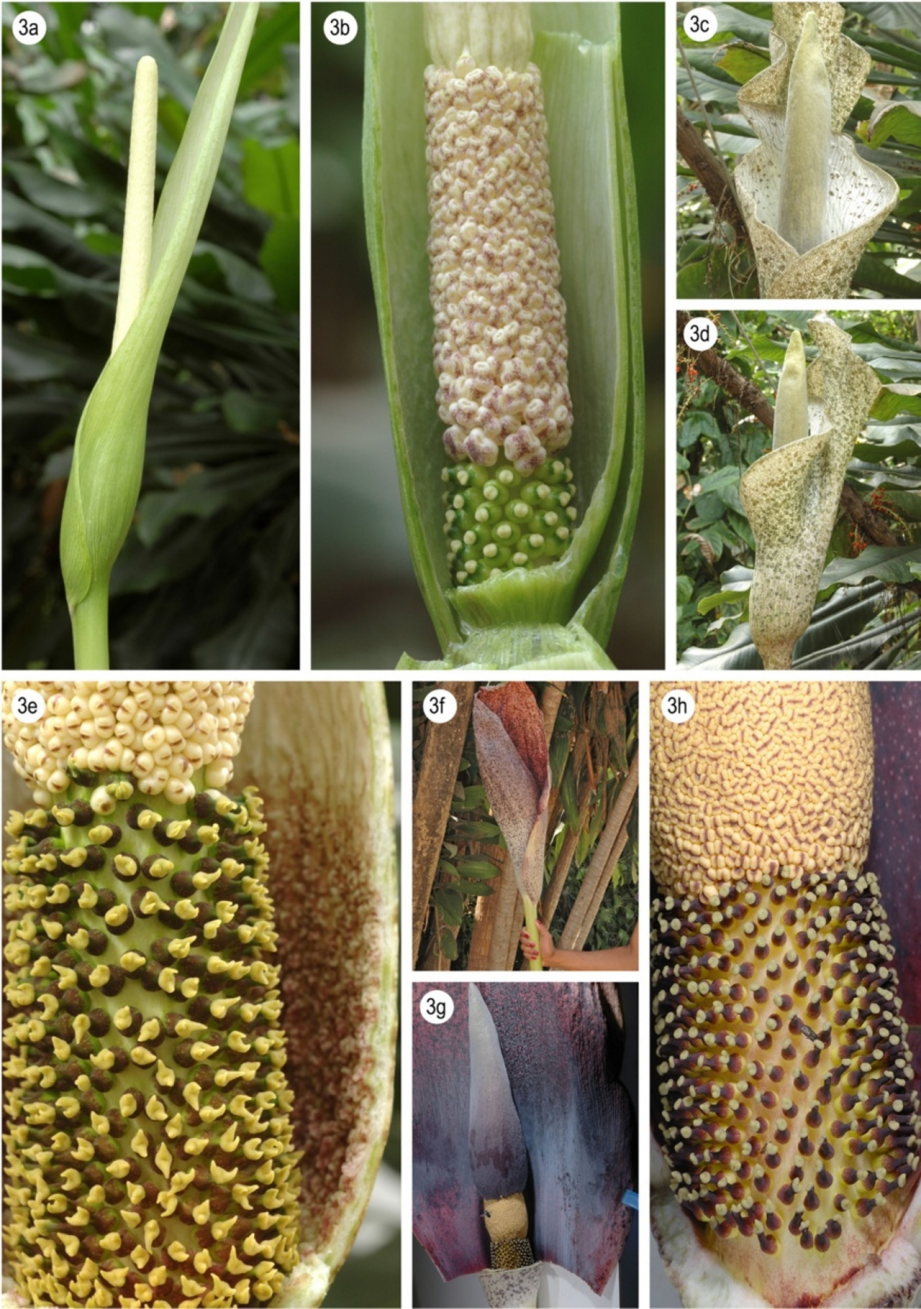
**Morphology of Amorphophallus species of Madagascar.** Amorphophallus antsingyensis, inflorescence **(a)**; Amorphophallus antsingyensis, detail of spadix base **(b)**; Amorphophallus erythrorrhachis, inflorescence, front view **(c)**; Amorphophallus erythrorrhachis, inflorescence, lateral view **(d)**; Amorphophallus erythrorrhachis, detail of spadix base **(e)**; Amorphophallus hildebrandtii, inflorescence **(f)**; Amorphophallus hildebrandtii, spathe cut open showing inner surface of the spathe and the entire spadix **(g)**; Amorphophallus hildebrandtii, detail of spadix base **(h)**. **f-h** by O. Pronk (Madagascar), others by W.L.A. Hetterscheid (Netherlands).

7a Ovaries (nearly all) bilocular, style absent, spathe entirely green, tuber disciform, offsets rhizomatous.....*A. antsingyensis* (Figure 4a, b)

7b Ovaries unilocular, style present and distinct, spathe maculate..8

8a Spathe opening wide, limb twisted helically or spreading, male zone longer than wide. .......................................................................................*A. erythrorrhachis* (Figure 4c-e)

8b Spathe for the greater part closed and tubular, limb very short, erect, male zone shorter than wide......*A. hildebrandtii* (Figure 4f-h).

### Amorphophallus perrieri

Hett. & Wahlert, **sp. nov.** Type: MADAGASCAR, Prov. Antsiranana: Nosy Ankarea, northwest of Nosy Mitsio, on top of island, 12°50'30“S 48°34'44”E, 220 m, 23 Nov. 2007, fl., *G. Wahlert 142*, (holotype P); Paratypes: MADAGASCAR, Prov. Antsiranana: Pan de Sucre, Nosy Mitsiou, X.1932, fl., *J.M.H.A. Perrier 18789* (P); same data as holotype, gathered on 1 Feb. 2012 from a flowering plant cultivated at the University of Utah greenhouse, Salt Lake City, UT, USA., *G. Wahlert 142bis* (MO); Nosy Ankarea, northwest of Nosy Mitsio, on top of the island, 12°50'30“S 48°34'44”E, 220 m, 23 Nov. 2007, *G. Wahlert & Rakotonasolo 42*, (HAST, TAN). (Figure 2a-h).

### Diagnosis

*Amorphophallus perrieri* is easily distinguished from its morphologically most similar congeners (*A. hildebrandtii*, *A. erythrorrhachis* and *A. taurostigma*, all from N. Madagascar) by the obtuse and heavily wrinkled/corrugated upper part of the appendix.

### Description

Seasonally dormant herb. Tuber depressed globose, 8–25 cm in diam. Leaves solitary, to 1 m tall, 1–1.2 m across; petiole: 50–0.84 m long, 2–7.5 cm in diameter at the base, 1–4 cm in diameter at the apex, smooth, spotted, white-pinkish or dirty pale greyish to ivory white background with oval to elliptic dark olive green spots with or without darker borders, spots often confluent; lamina decompound, rachises narrowly winged in the distal parts; leaflets elliptic-lanceolate to lanceolate, 2–14.5 cm long, 0.9 - 4 cm wide, (long) acuminate, upper surface mid green with or without a very narrow, pinkish border, lower surface pale green. Peduncle 50–105 cm long, 1.5 - 4 cm in diam. at the base, smooth, pale whitish greenish with numerous olive brown to olive green, elliptic to oval, partly confluent spots. Spathe triangular, 29–33 cm long, 14–16 cm in diam.; base tubular, strongly convolute, inside with few or numerous large warts arranged in longitudinal rows, surface white with a greenish flush, most basal part flushed with pale purple, upwards with increasing number of orbicular pale to dark maroon spots, outside whitish green with numerous orbicular or elliptical, partly or almost entirely confluent, brownish spots, often with a paler centre; limb erect or oblique, often rolling up over the dorsal surface, margin undulate-plicate, top broadly acute, inside pale or darker purple with numerous orbicular dark brownish-purple spots, more dense near the margin, veins sometimes conspicuously dark brown, outside as inside but slightly paler. Spadix as long as spathe or shorter or slightly longer, 22–23 cm long; female zone cylindric, 2.5 - 3 cm long, 1.8 - 2.5 cm in diam., flowers slightly distant; male zone more or less cylindric, 2–3 cm long, 1.9 - 2.3 cm in diam., flowers congested; appendix 16–17.5 cm long 3.4 - 3.8 cm in diam., lower half almost smooth or shallowly to more strongly corrugated or furrowed, upper half shallowly to strongly corrugated and folded and often inflated, top obtuse or irregularly truncated and furrowed, off white, yellowish white or pale green. Ovary globose or slightly ovoid, 2–2.5 mm long, 2 mm in diam., unilocular, white with a green flush, or bright green, with or without upper blackish purple part; style short and thick, conical or cylindrical, 1–1.2 mm long, 1 mm in diam., dark purple; stigma depressed or slightly conical or with one distinct, conical, obtuse or acute lobe, 1–1.3 mm in diam., 0.5 - 1 mm high, surface densely verruculate, off-white or dark greyish purple. Male flowers consisting of 4–5 stamens; stamens 2.5 mm long; filaments 0.5 mm long, partly confluent; anthers 2 mm long, 1.5 - 2 mm in diam., truncate, off-white, connective often greyish, pores apical. Infructescence elongate; berries ovate, colour changing from green to yellow to orange.

#### Etymology

The species is named after Joseph Marie Henry Alfred Perrier de la Bâthie (1873–1958) distinguished botanical explorer of Madagascar.

#### Distribution and ecology

*Amorphophallus perrieri* is known only from the islands of Nosy Ankarea, Nosy Mitsio and from the Kalabenono massif in the Antsiranana Province, northern Madagascar (Figure 1). Both areas are situated in the sub-humid forests of the Sambirano bioclimatic region (sensu Humbert 1955). At the type locality on Nosy Ankarea, the species is common in the understory of low stature, semi-deciduous forests on the top of the island and grows in rocky, well drained soils underlain by basalt. In the field, diptera were observed visiting the mature inflorescence, which has a pungent odour reminiscent of cheese, carrion, and faeces. The plants growing on Nosy Ankarea are likely to be protected well into the future. The local Sakalava ethnic group inhabiting the nearby island of Nosy Mitsio consider it *fady*, or taboo, for people to visit or inhabit Nosy Ankarea island because it is the ancestral burial ground of their ancient rulers (Wahlert, pers. comm.).

*Vernacular name* (Sakalava): “iriri”.

#### Additional specimens

*A. Dugger 0129*, Madagascar, Kalabenono, from a plant cultivated in the USA, 10 Oct. 2007 (WAG, spiritcoll; orig. coll. *O. Pronk’s collector s.n.*); *W.L.A. Hetterscheid H.AM.1601*. Madagascar, Kalabenono, from a plant flowering at the former Wageningen Bot. Garden, 6 Jan. 2008. (WAG, spiritcoll; orig. coll. *O. Pronk’s collector s.n*.); *W.L.A. Hetterscheid H.AM.1602*. Madagascar, Kalabenono, from a plant flowering at the Leiden Bot. Garden, 28 Febr. 2010. (L, spiritcoll; orig. coll. *O. Pronk’s collector s.n*.); *R.Kaufmann s.n.*, Madagascar, Kalabenono, from a plant cultivated in the USA, 2007 (WAG, spiritcoll.; orig. coll. *O. Pronk’s collector s.n.*); *G. Wahlert 143* (data as holotype; leaf parts only, CAS, G, HBG, K, NY, M, MO, P, WAG).

#### Notes

Species of this small group are indistinguishable in the vegetative state. The differences found in the inflorescences are never really impressive, with the exception of the short, conical appendix of *A. hildebrandtii* and the wrinkled/corrugated appendix top of *A. perrieri*. Thus far and however small, the differences (see also Table one in Hetterscheid & Mangelsdorff 2006) do seem to be stable. The fact that all of them are found in a relatively small part of Madagascar seems to indicate that they have only recently separated and evolved along their respective independent paths.

### *Amorphophallus hildebrandtii* revisited

The recent rediscovery of *A. hildebrandtii* by Prof. S. Vogel (in 1960) and Olaf Pronk (in 2007) and the subsequent successful cultivation of the species by the latter made it possible to update Engler’s description, that was based solely on the type specimen. As usual with *Amorphophallus* species, drying a specimen for herbarium purposes seriously deflates dimensions of many parts of the spadix, readily leading to misinterpretations of especially appendix shapes and dimensions, and similarly for pistils. Therefore this updated description of *A. hildebrandtii* is provided.

Seasonally dormant herb. Tuber depressed globose, at least to 25 cm in diam. Leaves solitary, to 2 m tall, 1–2 m across; petiole to 150 cm long, to 8 cm in diameter at the base, smooth, very variable, spotted, green with minute speckling or white-pinkish or dirty pale to dark greyish to ivory white background with oval to elliptic dark olive green spots with or without darker borders, spots often confluent; lamina decompound, rachises narrowly winged in the distal parts; leaflets elliptic-lanceolate to lanceolate, 2 - ca. 15 cm long, 0.9 - ca. 5 cm wide, acuminate, upper surface mid or pale green, somewhat glaucous, lower surface pale green. Peduncle to ca. 100 cm long, as petiole but usually paler. Spathe ovate, to 90 cm long, to ca. 70 cm in diam., base narrowly tubular, convolute, limb broadly tubular for the larger part of its length, erect, top slightly hooded, at first narrowly opening, later expanding more widely, base outside whitish, inside reddish purple with scattered, orbicular blackish spots, surface with a glaucous waxy layer, limb outside dirty brownish purple or pinkish purple with pale greenish main veins, slightly glossy, inside basal part uniformly blackish purple with a glaucous waxy layer, upwards reddish purple, with blackish veins and orbicular spots, the latter numerous and often confluent along the central part of the limb. Spadix sessile, distinctly shorter than spathe, 20 - ca. 40 cm long; female zone cylindric, 3.5 - 5 cm long, to 4 cm in diam., pistils not congested; male zone broadly cylindric-fusiform or obconical, 2–4 cm long, 3–4 cm in diam., stamens congested; appendix broadly to narrowly elongate conical, 10 - ca. 30 cm long, 4–6 cm in diam. at the base, top acute, surface very shallowly furrowed, blackish purple to dirty greenish. Ovary slightly elongate-ovate, 2.5 - 3 mm long, 2 mm in diam., unilocular, base pale greenish whitish, rest blackish purple; style distinct, slightly conical 1–1.5 mm long, 0.8 - 1 mm in diam., blackish purple; stigma depressed, obliquely inserted on the style, with one distinct, conical, obtuse or acute lobe, 1.3 mm in diam., 0.5 - 1 mm high, surface densely scabrate, off-white or dark greyish purple. Male flowers consisting of 4–5 stamens; stamens 3 mm long; filaments 1 mm long, only fused at the base; anthers 2 mm long, 1.5 - 2 mm in diam., truncate, orangish, connective often brownish, pores apical, slit-like.

Specimens studied: MADAGASCAR, Nosy Be, near Hellville, flowered 27 Oct. 2007 in cult. in Antananarivo, *O. Pronk s.n*. (WAG, spiritcoll., spadix only; orginal coll. *O. Pronk’s collector s.n.*); Same loc., flowered 30 Oct. 2007 in cultivation in Antananarivo, *O. Pronk s.n*. (WAG, spiritcoll., spadix only; orig. coll. *O. Pronk’s collector s.n.*).

### Phylogeny of endemic Madagascar *Amorphophallus* species

**Figure 5:**
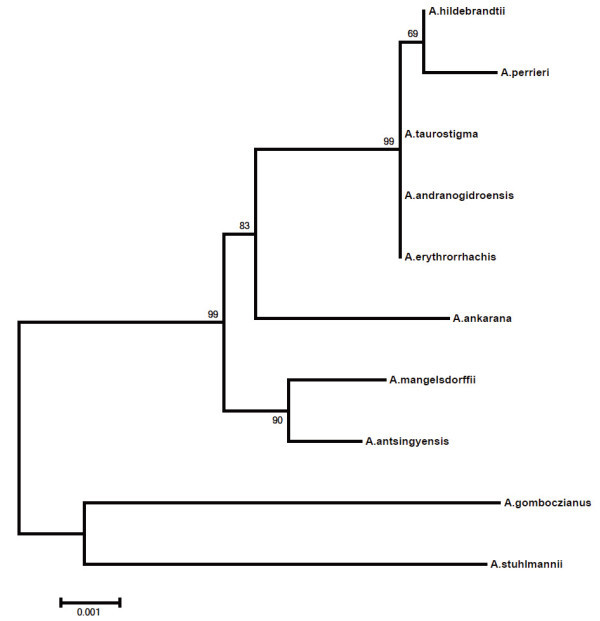
**Maximum-likelihood tree of the Madagascan species including two African species as outgroup (** ***A. gomboczianus*** **and** ***A stuhlmannii*** **).** Bootstrap values at the nodes.

The most recently published partial phylogeny of *Amorphophallus* (Sedayu et al. 2010) failed to retrieve the Madagascan endemic species as a monophyletic group. A phylogenetic investigation of the whole genus *Amorphophallus* based on four molecular markers (ITS1, Flint2, rbcL and matK) is currently being undertaken (Claudel et al. in prep.). As a preliminary result, the phylogeny of the eight Madagascan species only is presented here, including two African species (*A. gomboczianus* Pic. Serm. and *A. stuhlmannii* (Engl.) Engl. & Gehrm.) as outgroup species (see Figure 5).

The monophyly of the Madagascan species clade is very well supported with a bootstrap value of 99 encompassing two distinct groups. The first group contains *A. mangelsdorffii* + *A. antsingyensis* with a support of 90. Bogner (2003) suggested these two species to be more closely related to each other although their gross morphology does not support this. *Amorphophallus antsingyensis* actually looks quite like species from the other clade by its narrow spathe (Figure 4a), long rhizomatous offsets, lanceolate leaflets, distinct style, stigma shape (Figure 4b), etc. It is generally smaller in all its dimensions and almost uniformly green in all its parts but clearly the results presented here show such differences are not indicative of phylogenetic distance. *Amorphophallus mangelsdorffii* is in fact the oddity of all Madagascan species, owing to its deviating growth cycle, short, coiling spathe (Figure 3c), lack of differentiated style, and very small stigmas. The one character that does unite *A. mangelsdorffii* and *A. antsingyensis* is their spiny pollen (Bogner 2003, Figure 1; Hetterscheid et al. 1999, Figure 2). (Van der Ham et al. 2005), and as such may be regarded as a strong morphological synapomorphy between the two.

The second clade is less well supported (bootstrap 83) with the placement of *A. ankarana* as sister taxon to the remaining five species. These five, however, form a very well supported clade (bootstrap 99). In this clade *A. hildebrandtii* and *A. perrieri* form a weakly supported clade (bootstrap 69), whereas the relationship between the three remaining species (*A. taurostigma, A. andranogidroensis* and *A. erythrorrhachis*) is not resolved. Indeed these species are morphologically very similar. The main differences between them lie in the number of locules, and the shape and dimensions of the appendix. The variation of the appendix may be seen to indicate adaptations to different pollinators, and as such may indicate species distinctness. Variation in the number of locules in *Amorphophallus* in general shows three main patterns: stable unilocularity, stable bilocularity, and varying between two, three or four locules in one species. We suggest therefore that the difference between unilocularity and bilocularity in this group of Madagascan species has relevance in hypothesizing species distinctness.

## Conclusions

In this paper it was made clear that a new species of *Amorphophallus* exists in N. Madagascar which phylogenetically belongs to a smaller subclade of Madagascan species with similar morphologies. Phylogenetic analysis has confirmed that all endemic species of Amorphophallus on Madagascar belong to a strongly supported clade, contrary to the results of Sedayu et al. (2010). From this clade, the species *A. hildebrandtii* was shown to possess a very short spadix indeed unique to its clade, which was unclear from the only specimen known to date, the holotype in Berlin.

## Appendix

Mega parameters applied:

Analysis: Phylogeny Reconstruction

Statistical Method: Maximum Likelihood

Phylogeny Test: Bootstrap method

No. of Bootstrap Replications: 10000

Substitutions Type: Nucleotide

Model/Method: Kimura 2-parameter model

Rates among Sites: Uniform rates

Gaps/Missing Data Treatment: Complete deletion

Tree Inference Options: ML Heuristic Method, Subtree-Pruning-Regrafting - Extensive (SPR level 5)

Initial Tree for ML: Make initial tree automatically (Default - NJ/BioNJ)

Branch Swap Filter: Very Strong

Codons Included: 1st+2nd+3rd+Non-Coding

## Authors’ original submitted files for images

Below are the links to the authors’ original submitted files for images. Authors’ original file for figure 1 Authors’ original file for figure 2 Authors’ original file for figure 3 Authors’ original file for figure 4 Authors’ original file for figure 5
