# Supplementary figures and images for: Endemic Amorphophallus (Araceae) from Madagascar: a revised key, a new species and molecular phylogeny
Source: Bot Stud. 2014 Jan 14;55:2. doi: 10.1186/1999-3110-55-2 (PMC5432842; doi:10.1186/1999-3110-55-2)

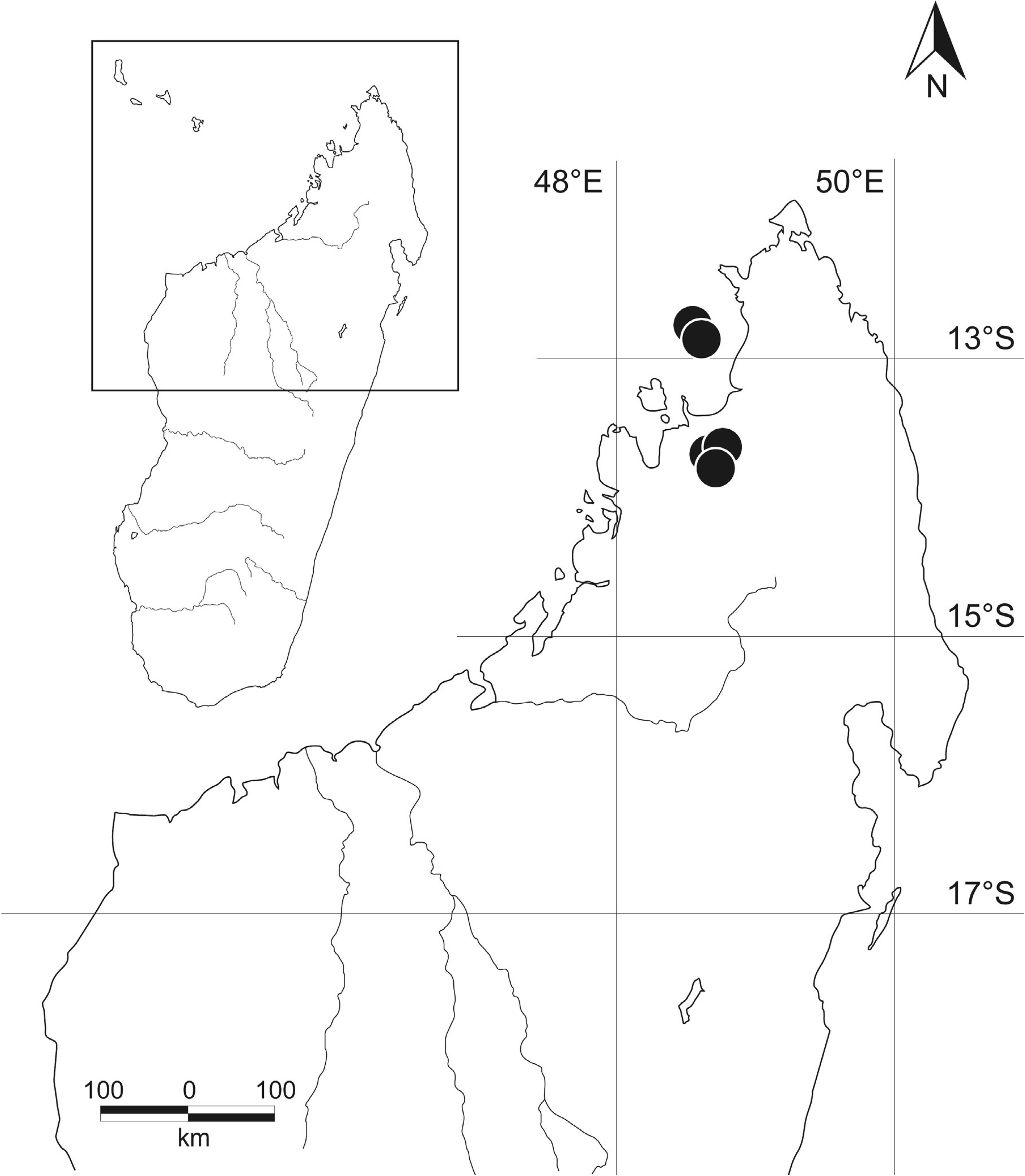

Supplement: Supplementary file 1 — Authors’ original file for figure 1 [file 40529_2013_59_MOESM1_ESM.tiff]

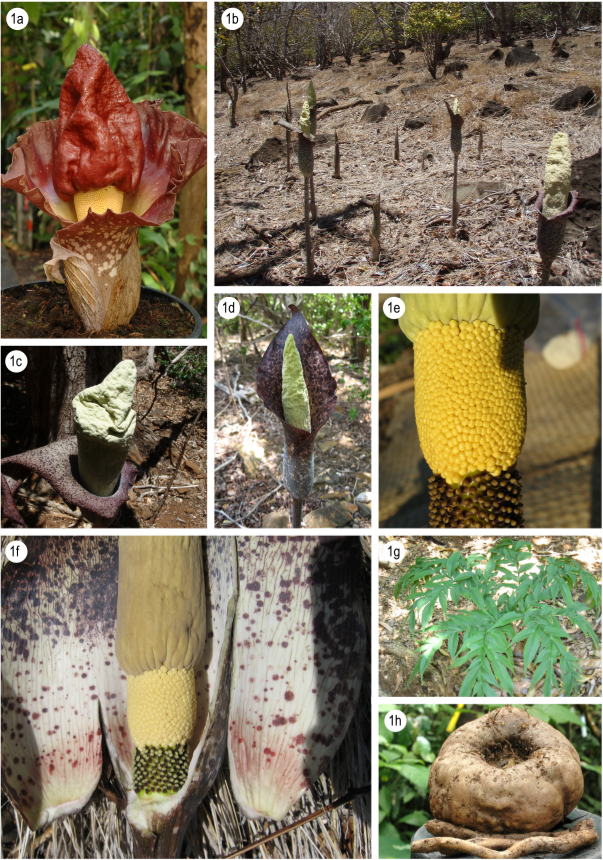

Supplement: Supplementary file 2 — Authors’ original file for figure 2 [file 40529_2013_59_MOESM2_ESM.png]

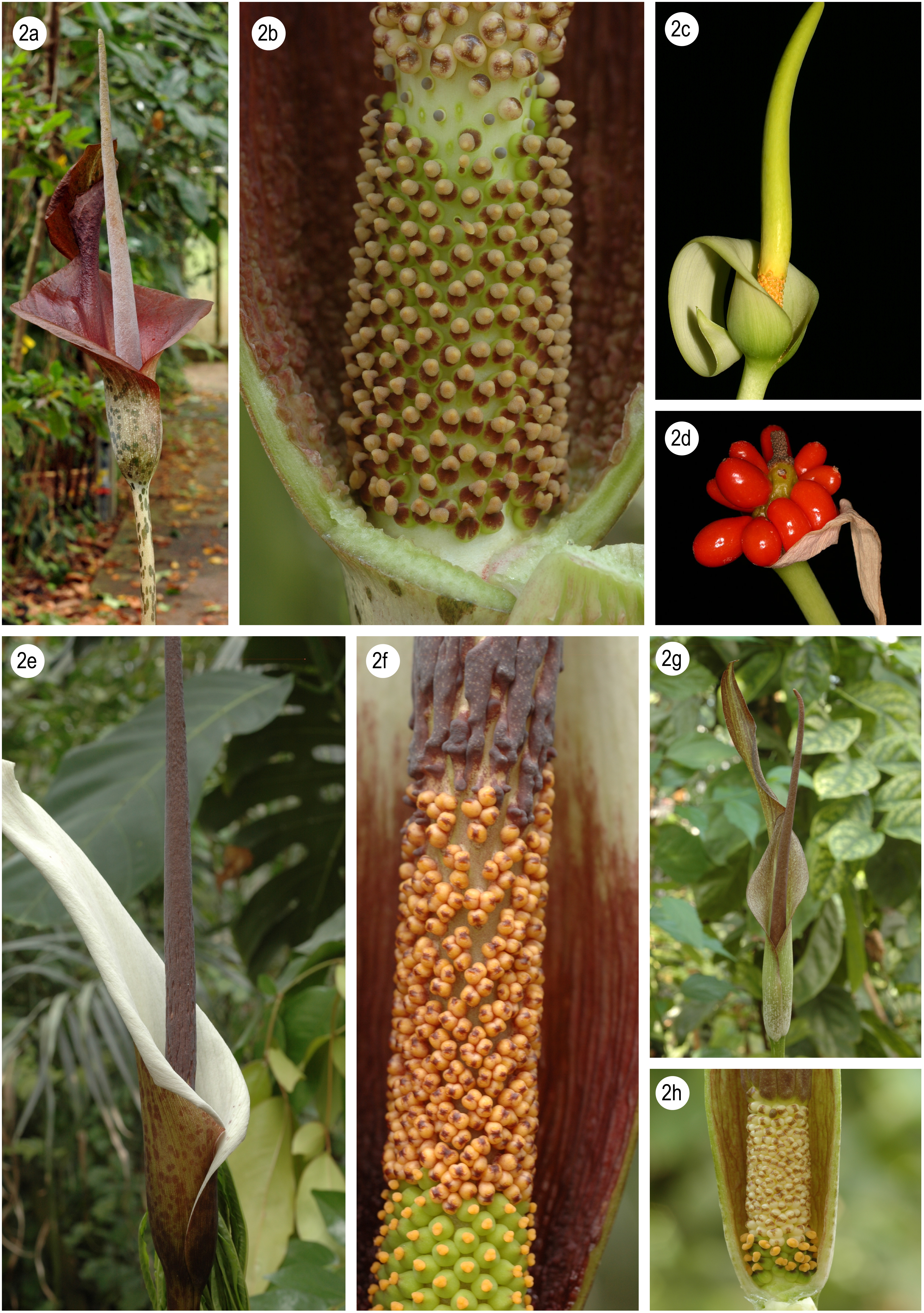

Supplement: Supplementary file 3 — Authors’ original file for figure 3 [file 40529_2013_59_MOESM3_ESM.jpeg]

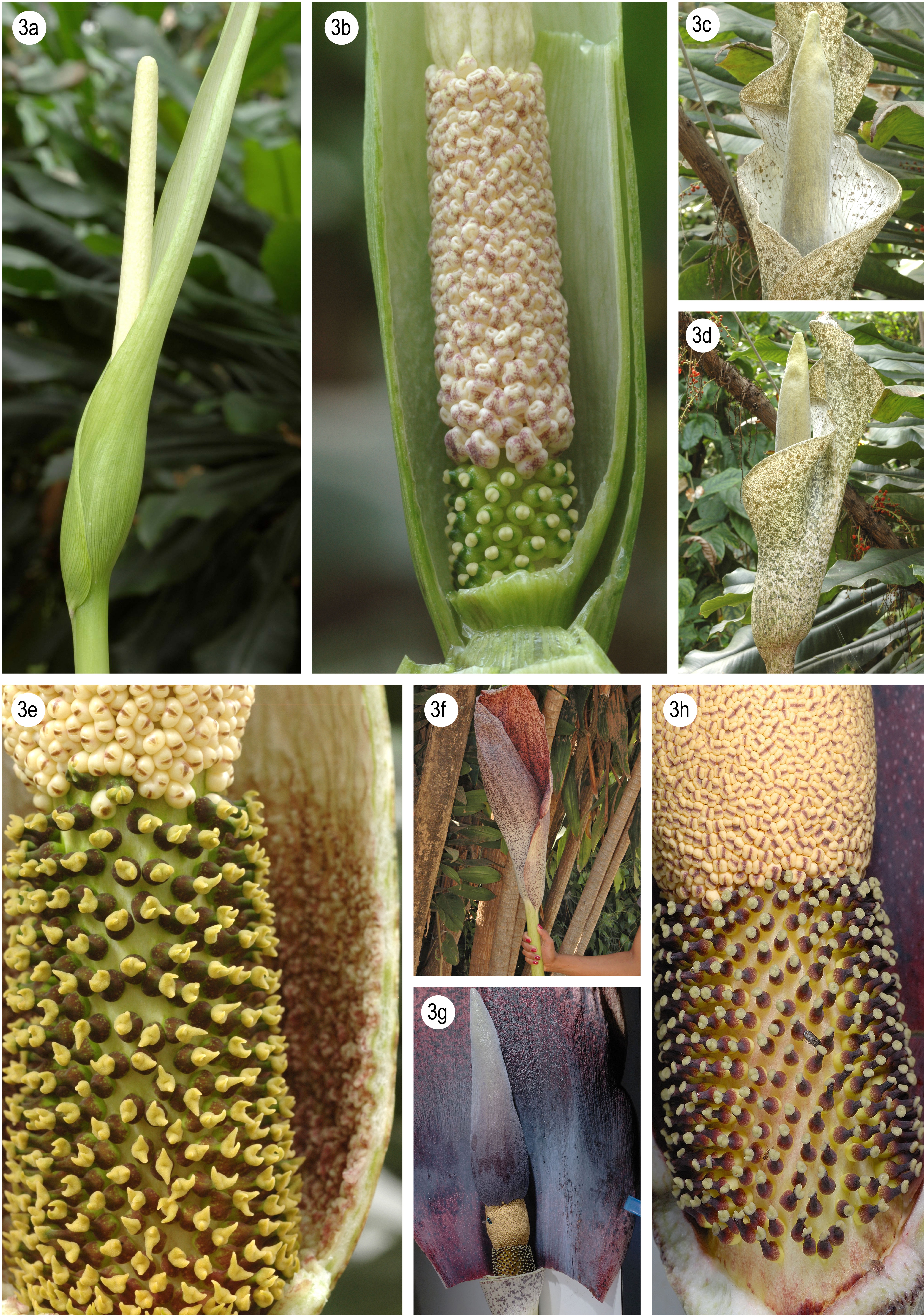

Supplement: Supplementary file 4 — Authors’ original file for figure 4 [file 40529_2013_59_MOESM4_ESM.jpeg]

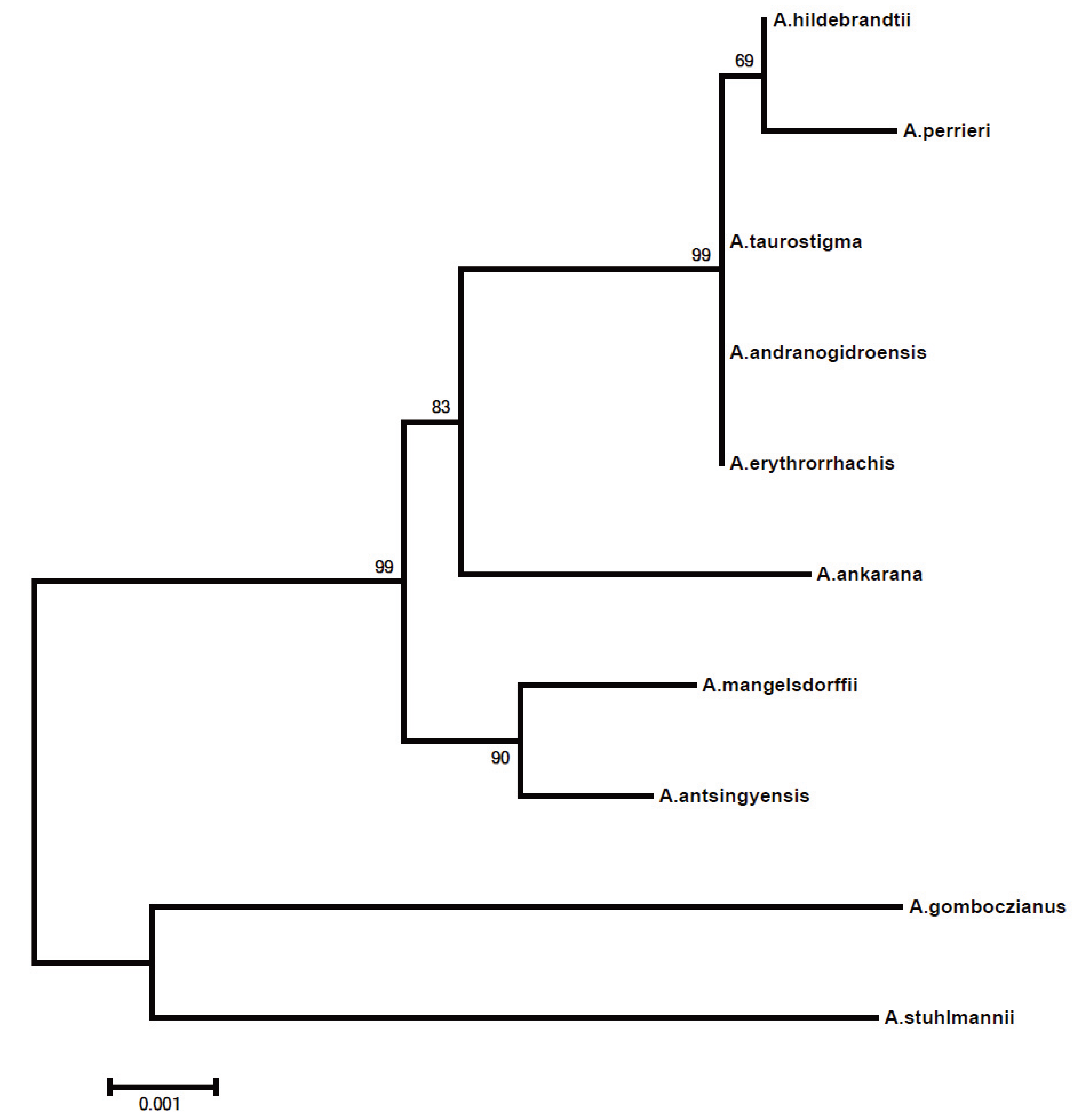

Supplement: Supplementary file 5 — Authors’ original file for figure 5 [file 40529_2013_59_MOESM5_ESM.tiff]
